# Fisher’s historic 1922 paper *On the dominance ratio*

**DOI:** 10.1093/genetics/iyac006

**Published:** 2022-03-03

**Authors:** Brian Charlesworth

**Affiliations:** Institute of Evolutionary Biology, School of Biological Sciences, University of Edinburgh, Edinburgh EH9 3FL, UK

**Keywords:** branching process, diffusion equation, dominance ratio, allele frequency distribution, heterozygote advantage, population size

## Abstract

R.A. Fisher’s 1922 paper *On the dominance ratio* has a strong claim to be the foundation paper for modern population genetics. It greatly influenced subsequent work by Haldane and Wright, and contributed 3 major innovations to the study of evolution at the genetic level. First, the introduction of a general model of selection at a single locus, which showed how variability could be maintained by heterozygote advantage. Second, the use of the branching process approach to show that a beneficial mutation has a substantial chance of loss from the population, even when the population size is extremely large. Third, the invention of the concept of a probability distribution of allele frequency, caused by random sampling of allele frequencies due to finite population size, and the first use of a diffusion equation to investigate the properties of such a distribution. Although Fisher was motivated by an inference that later turned out to lack strong empirical support (a substantial contribution of dominance to quantitative trait variability), and his use of a diffusion equation was marred by a technical mistake, the paper introduced concepts and methods that pervade much subsequent work in population genetics.

## Introduction

In 1922, R.A. Fisher published his paper *On the dominance ratio*, which can reasonably be thought of as the foundation paper for modern population genetics (Fisher 1922b). It influenced much subsequent work by J.B.S. Haldane and Sewall Wright, the other 2 “Founding Fathers” of theoretical population genetics, and made 3 major novel contributions to our understanding of genes in populations.

1. The formulation of a general model of selection on a single autosomal locus in an infinitely large population, and the discovery that selection can actively maintain variation in a population when there is heterozygote advantage at a locus with 2 alleles.
2. The discovery that a selectively favorable mutation has a substantial chance of loss from a very large population, and the use of the branching process for studying this problem quantitatively.
3. The invention of the concept of the probability distribution of an allele frequency, and the introduction of the diffusion equation method for analyzing the effects of random sampling of allele frequencies in a finite population.

Despite these important innovations, it attracted little attention for several years. Even J.B.S. Haldane, who was scrupulous about citing others, did not refer to it in his first 2 papers on the theory of selection (Haldane 1924a, 1924b).

To modern readers, the title of the paper may seem mystifying. Fisher’s main motivation was to examine the population genetic basis for what he believed to be an important general feature of the inheritance of quantitative traits, described in his famous 1918 paper. In that paper, he showed how the biometrical findings of Galton and Pearson on traits like human stature could be explained by the joint effects of multiple loci with individually small effects, together with nongenetic effects (Fisher 1918). He estimated that the dominance component of the genetic variance was often about 33% of the total genetic variance, and called this fraction the “dominance ratio.” This value came from the empirical observation that the correlation between mid-parent value and offspring value in human populations for traits like stature was smaller than the correlation between full siblings. To Fisher, this implied that a substantial component of variance is due to dominance, i.e. departures from additive effects on heterozygotes of the alleles at the individual loci contributing to variation. He wanted to find out why the dominance ratio had such a high value.

Fisher was also motivated by the proposal by the Dutch biologists, Arend Lourens Hagedoorn and Anna Cornelia Hagedoorn-Vorstheuvel La Brand, that random fluctuations in allele frequencies due to finite population size are of greater importance than selection in determining the level of variability in a species (Hagedoorn and La Brand 1921)—see Theunissen (2014) for an account of the Hagedoorns’ work in genetics and animal breeding. Fisher wrote a hostile review of their book (Fisher 1922a), starting with “*An excellent title is followed by a disappointing book*.” He went on to say “… *The authors believe… that the random selection of individuals to become the parents of the next generation is a more important factor in reducing the variability than is the natural selection of advantageous characters. … The whole question is worthy of a thorough discussion, but the authors evidently lack the statistical knowledge necessary for its adequate treatment*.” He then asserted that the rate of loss of variability caused by what he called the “*Hagedoorn effect*” [nowadays usually called “*random genetic drift*”; the term “*drift*” for this process was introduced by Wright (1931)], is the reciprocal of 4 times the population size, and he contrasted this with the rate of change in the frequency of a selectively favorable allele.

The introduction to the 1922 paper ended with the following remarks (pp. 323–324), which bring out the importance that Fisher attached to the dominance ratio. “*The decay in the variance of a species breeding at random without selection, and without mutation, is almost inconceivably slow: a moderate supply of fresh mutations will be sufficient to maintain the variability. When selection is at work even to the most trifling extent, the new mutations must be much more numerous in order to maintain equilibrium. That such is the case in mankind may be inferred from the fact that the frequency distribution of the numerical proportion of the allelomorphs, calculated on the assumption of selection maintained in equilibrium by occasional mutation, leads to the value of the Dominance Ratio which is actually observed*.”

As discussed below, Fisher’s belief that a dominance ratio of one-third reflects a fundamental property of the population genetic basis of quantitative trait variability was misplaced, and his analyses of the probability distributions of allele frequencies under different scenarios of selection and mutation were based on an erroneous formula, which was not corrected until several years later (Fisher 1930a). Nevertheless, the 1922 paper represents a major step forward in thinking about the causes of evolutionary change and natural variation. Indeed, the question of the nature of the population genetic processes maintaining variation in quantitative traits was first posed in this paper, and remains a very active research topic (Walsh and Lynch 2018, Chapter 28; Sella and Barton 2019).

## Fisher’s model of selection on a single locus, and the first analysis of balancing selection

Part 1 of the paper deals with equilibrium under selection acting on an autosomal locus, segregating for 2 alleles in an infinitely large, randomly mating, discrete-generation population. It was motivated by the fact that a loss of fitness under inbreeding is widely observed—Fisher cited a recently published book on inbreeding (East and Jones 1919). It had been known for several years that such an effect of inbreeding can be produced when heterozygotes at a biallelic locus have a higher fitness than the 2 homozygotes, but no population genetic analysis of this situation had been carried out.

Fisher used *a*, *b*, and *c* to denote the relative fitness of the 3 possible genotypes at an autosomal biallelic locus (A_1_A_1_, A_1_A_2_, and A_2_A_2_), and showed that only the cases when *b *>* a*, *c* or *b *<* a*, *c* permit an equilibrium with both alleles present in the population. He stated that the case *b *>* a*, *c* (heterozygote advantage) leads to a stable equilibrium of allele frequency, and that the opposite relation gives an unstable equilibrium, but did not formally prove these results. He suggested that the maintenance of variation by heterozygote advantage could cause hybrid vigor and inbreeding depression. “*Such factors should therefore commonly be found, and may explain instances of hybrid vigour, and to some extent the deleterious effects sometimes brought about by inbreeding.*” (Note that by “factor” Fisher meant a locus segregating for more than 1 allele.) The extent to which loci subject to heterozygote advantage contribute to inbreeding depression is still debated (Charlesworth and Willis 2009; Charlesworth 2015).

This discovery of Fisher’s was the first example of what came to be known as balancing selection, the active maintenance of variability by natural selection. Natural selection had initially been thought of in terms of one form replacing another, less fit, one. The first discovery of balancing selection was H.J. Muller’s analysis of a case of balanced lethals (Muller 1918); this involved 2 different arrangements of a *Drosophila* chromosome, inverted and standard arrangements, where both homozygotes were inviable because each arrangement carried a different recessive lethal mutation. Not until 1954 was the first example of heterozygote advantage at a single locus convincingly documented, in the classic case of sickle-cell hemoglobin (Allison 2004).

Fisher later introduced the concept of the maintenance of multiple phenotypic forms (morphs) by negative frequency-dependent selection in systems of Batesian mimicry (Fisher 1925, 1930b, Chapter 7). Many other types of balancing selection are now recognized (Charlesworth and Charlesworth 2010, Chapter 2), and searches for signatures of balancing selection are an important part of modern population genomics research (Fijarczyk and Babik 2015).

## The survival of a new mutation

In part 2, Fisher initiated the analysis of stochastic effects on allele frequencies by examining the fate of a mutant gene in a very large, discrete-generation population. Fisher realized that, when a mutation has newly arisen and is very rare, its carriers can be studied in isolation from the rest of the population, either in the case of a haploid population, or the heterozygous carriers of a mutant allele in a randomly mating, diploid population. In both cases, the mutant allele is initially transmitted clonally. In the simplest situation, the population itself is assumed to be stationary in size, so that the expected number of successful offspring per carrier of a neutral mutation is 1. Denoting the number of offspring for carriers of a favorable mutation by *m*, the case when *m *>* *1 implies that the mutation is favored by selection, with a selective advantage of *s *=* m* − 1.

Fisher treated the problem by using a probability-generating function (p.g.f.), a concept that traces back to De Moivre in the 18th century. He assumed that there is random variation among individuals of the same genotype, such that *p_i_* is the probability that *i* offspring are contributed by an individual to the next generation (usually defined as the interval from zygote to zygote). The fact that each carrier of a mutation gives rise to an uncertain number of descendants is the essence of the branching process, whose study long predates Fisher’s application of it to genetics, although it is unclear whether Fisher was aware of this (Kendall 1966; Bru et al. 1992).

The p.g.f. is defined as:

(1) $$f\left(x\right)=p_{0}+xp_{1}+x^{2}p_{2}+..+{\;x}^{i}p_{i}+\ldots$$

If there were only a single copy of the mutant gene in the first generation after its occurrence, the probability that there are *i* copies in the next generation is the coefficient of *x^i^* in the p.g.f. After 1 generation, the generating function becomes *f*(*f*(*x*)), after another generation *f*(*f*(*f*(*x*))), and so on. This provides a useful method of iterating the probability distribution of the numbers of copies of the mutation over the generations. However, once the mutation becomes sufficiently common, the rest of the population cannot be ignored and the method is no longer applicable, but by then the mutation is unlikely to be lost from the population and its spread can be treated deterministically.

Fisher proposed that it is biologically realistic to assume that the offspring number of individuals follows a Poisson distribution with mean *m*, because the chance that a given offspring individual comes from a specified parent is very small when the population size is large. In this case, the p.g.f. has the form exp [*m*(*x* − 1)], and *p*_0_ = exp (−*m*), which is the chance of loss in the first generation after the appearance of the mutation. If *m *=* *1.10 (i.e. there is a 10% selective advantage), *p*_0_ = 0.333. Fisher asserted that, for a neutral mutation with *m *=* *1, there is only a 2% chance that it will survive 100 generations, and that the survivors will be represented on average in 50 individuals. He concluded by writing:

*“Only when the number of individuals affected becomes large will the effect of selection predominate over that of random survival, though even then only a very small minority of the population may be affected.”*

This aspect of Fisher’s paper attracted the attention of J.B.S. Haldane, who wrote a famous paper that considerably extended Fisher’s treatment (Haldane 1927). In particular, Haldane realized that the probability of ultimate loss of a mutation is given by the smaller solution with 0 ≤ *x *≤* *1 of the equation *x *=* f*(*x*). This result had been found previously by the French mathematician I.J. Bienaymé in 1845, in the context of the survival of human family names (Bru et al. 1992). In the case of a Poisson distribution of offspring number, and a value of *s* that is sufficiently small that *s*^2^ is negligible compared with *s*, this formula leads to Haldane’s well-known approximation 2*s* for the ultimate survival probability of a weakly selected mutation.

It is often a surprise to students to learn that a mutation with a selective advantage of, say, 10% has an approximately 80% chance of being lost from a large population. One important implication is that different populations exposed to the same selection pressure may adapt by establishing mutations at different nucleotide sites or in different genes, since it is a matter of chance which beneficial mutation will succeed in a given population (Mani and Clarke 1990; Charlesworth and Charlesworth 2010, Chapter 3; Ralph and Coop 2010); human resistance to malaria is an example of such a multiplicity of independent genetic responses to the same selection pressure (Kwiatkowski 2005).

In his 1930 paper, much of which is reproduced in Chapters 4 and 5 of *The Genetical Theory of Natural Selection* (*GTNS* for short), Fisher made elegant use of the branching process method to study the probability distribution of allele frequencies for allele frequencies near zero or 1 (Fisher 1930a, 1930b). He also provided an independent derivation of Haldane’s approximation for the survival probability of a mutation, using the diffusion equation method discussed below. These results on survival probabilities have been very widely used in evolutionary genetics, especially in models of molecular evolution (Kimura and Ohta 1971; Kimura 1983), the theory of adaptive walks (Orr 2005; Walsh and Lynch 2018, Chapter 27), and models of selective sweeps (Walsh and Lynch 2018, Chapter 10; Stephan 2019).

## The effects of finite population size on neutral variability

This section of the paper introduced the mathematical study of stochastic changes in allele frequencies due to finite population size into evolutionary biology. Fisher used what is now often called the “*Wright-Fisher model*” (Ewens 2004, p. 21), in which a discrete-generation population of *n* adult individuals is assumed to follow a binomial sampling process with respect to the allele frequency at a diploid, biallelic locus. If the frequencies of alleles A_1_ and A_2_ are *p* and *q *=* *1 − *p*, respectively, the variance of *p* after 1 generation is *pq*/(2*n*). This dependence on *p* is a complication, which Fisher wished to avoid. He used the trick of transforming to a variable *θ* such that cos(*θ*) = 1 − 2*p*; with *p* varying from 0 to 1, cos(*θ*) lies between 1 and −1, and *θ* and 0 ≤ *θ* ≤ *π* (where *θ* is measured in radians). On this scale, the variance of *θ* due to 1 generation of sampling is approximately 1/(2*n*); the error in this approximation is negligible for the large values of *n* assumed by Fisher.

The underlying assumption is that the process of random sampling creates a frequency distribution representing the behavior of a large number of loci. These loci are all initially segregating for pairs of alleles at the same frequencies. They are then allowed to evolve independently under random sampling each generation, so that the allele frequencies at different loci diverge; in each generation, there is a nonzero chance that a given locus become fixed for one or other allele. Fisher was especially interested in the rate at which loci become fixed and hence lose variability, and in the mean number of loci that remain segregating. This is obviously relevant to the Hagedoorns’ ideas, mentioned in the Introduction.

To analyze this problem, Fisher assumed that the population size *n* is sufficiently large that, to a good approximation, *p* can be treated as a continuous variable; in reality, *p* varies between 0 and 1 in increments of 1/(2*n*). The probability density function (p.d.f.) for *θ* in generation *T* can be written as *y*(*θ, Τ*). Fisher assumed a normal distribution of changes in *θ* due to sampling over 1 generation (p. 327), and set up an integral equation for the value of *y*(*θ, Τ*) in the next generation by assuming that changes per generation in *θ* are small (normality need not be assumed; it is sufficient to neglect third- and higher-order powers of the changes, e.g. Kimura 1964).

This procedure yielded the following partial differential equation for *y*(*θ, Τ*) in the case of neutrality, where *T* is time measured in generations:

(2) $$\frac{\partial y}{\partial T}=\frac{1}{4n}\frac{\partial^{2}y}{\partial \theta^{2}}$$

This is the first example of the use of a diffusion equation in population genetics, and is identical in form to the equation derived by Einstein to describe Brownian motion (Einstein 1905). Following the example of Wright (1945), diffusion equations have become a powerful tool for theoretical population geneticists, especially in the hands of Warren Ewens, Motoo Kimura, and Tomoko Ohta (Kimura 1964, 1983; Kimura and Ohta 1971; Ewens 2004).

Equation (2) tacitly assumes that the mean change of *θ* between 2 generations, conditioned on a given value of *θ*, is zero; this is an error, as discussed below. By a rather complex argument, Fisher concluded that the rate of change per generation of *y* is asymptotically −*y*/(4*n*) (p. 330). This result can be reached more easily: assume that *y* reaches an asymptotic state such that the left-hand side of Equation (2) is equal to −*ky*, where *k* is a positive constant. There is then a constant proportional rate of decline in the probability density of a given *θ* value, due to fixation of one of the 2 alleles at a segregating locus, and Equation (2) gives the following equation:

(3) $$\frac{\partial^{2}y}{\partial \theta^{2}}=-\frac{1}{4n}ky$$

This property is expected on general principles, but was only formally derived in 1955 by the use of a complex general expression for the p.d.f. of the allele frequency itself (Kimura 1955). McKane and Waxman (2007) have extended the treatment of this case to include the frequencies of the 2 fixed classes as well as the p.d.f. for the segregating loci.

Assume that *y*(*θ, T*) can be expressed as the product of a function of *T* and a function of *θ*, as is usual in treatments of this type of equation (Kimura 1955; Ewens 2004, p. 151, 159). Equations (2) and (3) then have the solution *y*(*θ*, *T*) = *A* sin(*θ*) exp(−*kT*), with *k *=* *1/(4*n*), where *A* is a constant determined by the initial value of *θ*. The term sin(*θ*) can be interpreted as the p.d.f. for *θ* at segregating loci. This implies that, in the long run, the proportion of segregating loci declines at a rate of 1/4*n.*

Fisher believed that *n* is likely to be in the millions for most species so that the rate is negligibly small, contradicting the Hagedoorns’ argument (p. 330). “*This is a very slow rate of diminution, a population of* n *individuals breeding at random would require* 4n *generations to reduce its variance in the ratio of 1 to* e*, or 2.8 to halve it. As few specific groups have less than 10,000 individuals between whom interbreeding takes place, the period required for the Hagedoorn effect, in the entire absence of mutation, is immense*.”

## Fisher’s error

The error in Equations (2) and (3) came to light in the following way. According to William Provine’s biography of Sewall Wright (Provine 1986, Chapter 8), Fisher met Wright when he was on a tour of the USA in 1924, having been impressed by Wright’s 1921 papers on inbreeding and quantitative inheritance (Wright 1921a, 1921b). He later sent Wright a copy of the 1922 paper, which stimulated Wright to start to think about evolution in terms of population genetics. By 1925, Wright had written a lengthy manuscript, which evolved into his classic paper *Evolution in Mendelian populations* (Wright 1931). He assumed the same model as Fisher, and used his path coefficient method to deduce the rate of decline in 1 – *F* (where *F* is the inbreeding coefficient), finding it to be 1/(2*n*), not 1/(4*n*). Wright struggled to understand where the discrepancy came from, assuming that the rate of decline in 1 – *F* should be the same as Fisher’s rate of loss of segregating loci. He also used an alternative method of handling the distribution of allele frequencies (without using a transform), and found the rate of loss of segregating loci to be 1/(2*n*).

As a result of correspondence with Wright about this problem, Fisher realized that he was mistaken in assuming that a mean change in *θ* over 1 generation under pure random sampling is zero; this is true of the allele frequency itself, but the correct expression for the mean change in *θ* is—*y* cot(*θ*)/4*n*. The full differential equation for *y* when there is a mean change in *θ* per generation of *Μ_δθ_* is:

(4) $$\frac{\partial y}{\partial T}=-\frac{\partial \left(yM_{\delta \theta }\right)}{\partial \theta }+\;\frac{1}{4n}\frac{\partial^{2}y}{\partial \theta^{2}}\;$$

Neither Fisher nor Wright realized that this expression is an example of the equation already known to physicists as the Fokker–Planck equation, which was discovered independently by Adriaan Fokker in 1914 and Max Planck in 1917 (Fokker 1914; Planck 1917); Wright was informed of this much later by a colleague (Wright 1949). It was also found independently by the eminent Russian mathematician A. N. Kolmogorov in 1931 (Kolmogorov 1931), and is often referred to as the “Kolmogorov forward equation.” Kolmogorov himself used it for solving a population genetics problem (Kolmogorov 1935). He later wrote to Wright, who published a paper applying it to the allele frequency rather than to Fisher’s transform (Wright 1945). This method has now become standard practice in population genetics (Kimura 1964; Ewens 2004, Chapters 4 and 5; Charlesworth and Charlesworth 2010, Chapters 5, 6, and 8; Walsh and Lynch 2018, Appendix A1). The use of the transform to *θ* now seems like a distraction (and the use of the transform certainly contributes to the difficulty in following Fisher’s calculations).

In 1930, Fisher revised his 1922 calculations; for the case of pure drift, he showed that *y* is still proportional to sin*θ*, but that the (asymptotic) rate of loss of segregating loci is equal to Wright’s value of 1/(2*n*) (Fisher 1930a). Using the rule for a change of variable in a p.d.f., it is straightforward to show that *y *=* A* sin*θ* transforms to a uniform distribution of allele frequency *p*, in agreement with Wright’s analysis (Wright 1931). Fisher preferred to work with the logit transform, *z* = ln(*p*/*q*), which ranges from *z* = −∞ to *z* = ∞. For the case of a constant rate of loss of segregating loci, this gives the curve in Figure 1 of Fisher (1922b). Kimura later showed that it can take a very long time (of the order of 4*n* generations) to reach this state (Kimura 1955), so that this result is of limited value as far as biology is concerned. In contrast, and in agreement with Wright’s finding using the inbreeding coefficient, the mean heterozygosity at a locus, 2*pq*, always declines at a constant proportional rate of 1 − 1/(2*n*).

It is interesting to note that Kolmogorov had great respect for Fisher’s 1930 work. In an anecdote related by Kendall (1990), at a 1967 conference on branching process theory, Kolmogorov was irritated by the highly abstract work presented by several mathematicians, and decided to remind them of the biological origins of the subject, referring to the *GTNS* as “*das wundervolle Buch von R.A. Fisher* [the wonderful book of R.A. Fisher].” Kendall wrote that “*Two United States mathematicians sitting near to me were heard to whisper* ‘*It* can’t *be* the *R.A. Fisher we know*’. *There is another half to that story. Will Feller used to say that if Kolmogorov had not written his 1931 paper, the whole of stochastic diffusion theory would have eventually been pieced together starting with the ideas in Fisher’s book.*”

Fisher used his 1922 formula for *y* to calculate the expected dominance ratio given by the distribution of allele frequencies, using his 1918 formulae for the additive and dominance variances (denoted here by *V_A_* and *V_D_*), and assuming complete dominance of one or other allele at a given locus. As was done by Wright (1931), it is easier to work directly with the p.d.f. for allele frequency *p*, *ϕ*(*p*), and to integrate the products of *V_A_* and *V_D_* with *ϕ*(*p*) over the uniform distribution of *p* between 0 and 1 at segregating loci. For a single biallelic locus with allele frequency *p*, writing the difference between homozygotes A_1_A_1_ and A_2_A_2_ as 2*a*_1_, and the difference between the heterozygotes’ value and the mean of the 2 homozygotes as *d*, we have *V_A_* = 2*a*_1_^2^*pq* [*a*_1_ + *d*(*q* − *p*)]^2^ and *V_D_* = 4*d*^2^*p*^2^*q*^2^. With complete dominance (*d* = *a*_1_), *V_A_* = 8*a*_1_^2^*pq*^3^ and *V_D_* = 4*a*_1_^2^*p*^2^*q*^2^. Integrating these expressions to obtain the mean values, *V_AM_* and *V_DM_*, where *M* denotes a mean, we have *V_AM_* = 2*a*_1_^2^/5 and *V_DM_* = 2*a*_1_^2^/15, so that *V_DM_*/(*V_AM_* +*V_DM_*) = 1/4, as was found by Fisher.

Fisher also considered the stationary neutral case when the loss of variability due to sampling is balanced by rare mutations, assumed to be equally frequent in each direction. In this case, the derivative of the frequency distribution is set equal to zero, and the solution for Fisher’s (incorrect) 1922 diffusion equation is a uniform distribution in *θ*. Using the formula for the p.d.f. of a transformed variable, the p.d.f. for *p* is proportional to 1/√(*pq*). This is wrong, for the reasons described above, and the correct distribution for *p* is a beta distribution (Wright 1931). With mutations in each direction at rate *u*, and writing 4*nu* = *U*, the distribution has the form:

(5) $$\phi \left(p\right)=\;\frac{{\Gamma (U)}^{2}}{\Gamma (2U)}p^{U-1}\;q^{U-1}$$

where Γ(*U*) is the gamma function (for a derivation, see Charlesworth and Charlesworth 2010, pp. 233–234). The moments of the beta distribution are well known, so it easy to obtain expressions for *V_AM_* and *V_DM_*.

When *U ≪* 1, which is the most likely situation for a single genetic locus given that mutation rates are generally very small compared with 1/*n* (Walsh and Lynch 2018, p. 106), *ϕ*(*p*) is inversely proportional to *pq*, so that most allele frequencies at segregating loci are close to 0 or 1. Assuming complete dominance of one or other of the 2 alleles, we then have:

(6) $$V_{AM}=\frac{8}{\;3}\;a_{1}^{2}\;U;V_{DM}=\frac{2}{\;3}\;a_{1}^{2}U;\;\frac{V_{DM}}{V_{AM}+V_{DM}}=0.\text{2}$$

This dominance ratio of 0.2 is close to Fisher’s value of 0.2308.

## The effects of selection

Fisher also considered the effects of directional selection at a biallelic locus. He first considered what he called “*uniform genetic selection*”: the heterozygotes’ fitness is the geometric mean of the homozygotes’ fitnesses, i.e. it is the square root of the product of the homozygotes’ fitnesses. If *a*, *b*, and *c* are the relative fitness of the 3 genotypes, A_1_A_1_, A_1_A_2_, A_2_A_2_, we can write *r *=* a*/*b *=* b*/*c*. The recurrence relationship for allele frequency then has an explicit solution. If *p_t_* is the frequency of allele A_1_ in generation *t* and there is random mating, we have:

(7) $$\frac{p_{t}}{q_{t}}=r^{t}\frac{p_{0}}{q_{0}}$$

Fisher assumed that *r* is close to 1, and used *α* = ln (*r*) as a measure of the strength of selection (this is approximately the same as assuming that the heterozygote is exactly intermediate in fitness between the homozygotes, i.e. there is semidominance with respect to fitness). The mean change in allele frequency per generation is now approximately equal to *αpq*, which can be plugged into the diffusion Equation (4) after a change of variable to *θ*.

Fisher analyzed the distribution of allele frequencies under this model, and evaluated the dominance ratio; given his erroneous assumption about the diffusion equation, this approach was necessarily flawed. However, the correct distribution under drift, mutation and semidominant selection was obtained by Wright (1931), and has the form:

(8) $$\phi \left(p\right)=C\;\exp\left(4n\alpha p\right)p^{U-1}q^{U-1}$$

where *C* is a constant that ensures that the distribution integrates to 1.

Even for this case, the expressions for the moments for arbitrary 4*nα* are quite complicated, involving confluent hypergeometric functions (Kimura et al. 1963; Charlesworth and Jain 2014). However, if selection on individual loci is very weak, as may reasonably be supposed to be true for a quantitative trait controlled by many loci, this can be approximated by a beta distribution similar to that in Equation (5) by setting the exponential term to one, which reduces to the previous neutral case with a dominance ratio of 0.2.

If selection is strong, so that 4*nα* ≫ 1, the distribution can be approximated by another beta distribution, assuming that deviations from the mean of *p* are small (Charlesworth and Charlesworth 2010, p. 354). In this case, the mean frequency of the selectively disadvantageous allele A_2_ is approximately the same as the infinite population equilibrium value, q* = *u*/*α*, with q* ≪ 1 (Haldane 1927). If each selected locus affects a quantitative trait, with a difference of 2*a*_1_ between the 2 homozygotes as before, then the assumption that there are equal frequencies of loci with dominant and recessive effects (as was done by Fisher) gives:

(9) $$V_{AM}\approx 4a_{1}^{2}u/\alpha ;V_{DM}\approx 4a_{1}^{2}u/n\alpha$$

The dominance ratio in this case is close to zero, since *V_DM_* ≪ *V_AM_* if *n* ≫ 1.

Fisher (p. 334) made some interesting remarks concerning variability under selection and drift, which are qualitatively correct and were followed up in his 1930 paper and book (Fisher 1930a, 1930b). “*The existence of even the slightest selection is in large populations of more influence in keeping variability in check than random survival. A further effect of selection is to remove preferentially those factors* [segregating loci] *for which α is high, and to leave a predominating number in which α is low. In any factor α may be low for one of two reasons: (1) the effect of the factor on development may be very slight, or (2) the factor may effect changes of little adaptive importance. It is therefore to be expected that large and easily recognised factors in natural organisms will be of little adaptive importance, and that the factors affecting important adaptations will be individually of very slight effect. We should thus expect that variation in organs of adaptive importance should be due to numerous factors, which individually are difficult to detect.*”

Fisher also considered the case of what he called “uniform genotypic selection,” i.e. complete dominance in the fitness effect of A_1_, so that the relative fitnesses of A_1_A_1_, A_1_A_2_, A_2_A_2_ are *a *=* b *≠* c*. He showed that the recurrence relation is then:

(10) $$\frac{p_{t\;}}{q_{t}}=\frac{p_{t-1}}{q_{t-1}}\frac{a}{ap_{n-1}+c}\approx \frac{p_{t-1}}{q_{t-1}}\;(1+q_{t-1}\beta )$$

where *β* = (*a*/*c*) − 1, which is what we would now call a selection coefficient (pp. 334–335).

He went on to remark that “*Genotypic selection resembles genetic selection in diminishing the amount of variability which a given frequency of mutation can maintain… it differs, however, in being comparatively inactive in respect of factors in which the dominant allelomorph is in excess, and consequently in allowing a far greater number of factors to exist in this region.*” The inefficiency of selection against deleterious recessive alleles had previously been pointed out by R.C. Punnett (1917) in an argument against eugenics, using a calculation supplied by the mathematician G.H. Hardy, and was later strongly emphasized by Haldane (1924a, 1927). Fisher (1924) later countered Punnett’s argument by pointing out that it is not necessarily true that traits such as mental disability are simple Mendelian traits, as was assumed by Punnett [for a discussion of Fisher’s view on eugenics, see Bodmer et al. (2021)].

To calculate the dominance ratio, Fisher assumed that the effects of the alleles at each locus on the trait are unidirectionally dominant with respect to increases in the trait value (A_1_ is dominant over A_2_), and that the recessive alleles at each locus are selected against according to the genotypic selection model with *β* > 0. Fisher’s treatment of the distribution of allele frequency using *θ* was, of course, flawed, so that his quantitative treatment is inaccurate. He also briefly analyzed assortative mating with respect to a quantitative trait and concluded that it would have little effect on the dominance ratio.

To do this, he studied the dynamics of selection under this model, and the resulting probability distribution of allele frequencies, assuming that the assortative mating causes negligible deviations from Hardy–Weinberg proportions at individual loci.

The correct distribution of allele frequencies under weak selection with arbitrary dominance and random mating was not found until 1937 (Wright 1937). For complete dominance and symmetrical mutation, we have:

(11) $$\phi \left(p\right)=C\;\exp\left(-2n\beta q^{2}\right)p^{U-1}q^{U-1}$$

Once again, the moments of this distribution do not have a simple form, so that it is not possible to get simple and general expressions for the variance components. However, some insights can be obtained without exact results. With very weak selection, this case reduces to the neutral one already considered, with a dominance ratio of 0.2. But, as Fisher noted, selection against deleterious recessives means that the distribution is more skewed to low values of *q* than under neutrality. With complete dominance of A_1_ alleles, loci with low *q* contribute more to *V_D_* than to *V_A_*, so we expect a larger dominance ratio than under neutrality. In the limiting case of 2*nβ* ≫ 1, the mean of *q* ≈ √(*u/β*) (the deterministic value) and deviations from it can be neglected. This gives:

(12) $$V_{AM}\approx 8{\left(\frac{u}{\beta }\right)}^{\frac{3}{2}}a_{1}^{2};\;V_{DM}\approx \;4\;\left(\frac{u}{\beta }\right)a_{1}^{2}$$

The dominance ratio therefore approaches one, since $V_{DM}/V_{AM}\approx \frac{1}{2}\sqrt{\beta /u}$ ≫ 1.

## Conclusions about the dominance ratio

Fisher was correct in thinking that there are circumstances under which the dominance ratio can be substantial when selection is sufficiently weak that drift has a substantial effect on allele frequencies, or with unidirectional dominance and selection against recessive alleles. Not surprisingly, Fisher asserted (p. 337) that the latter is the case “*which most nearly reproduces natural conditions*.” He summarized (p. 338) by writing: “*In the light of the above discussion in which we have deduced the distribution of allelomorphic ratios from the conditions of equilibrium with selective influences from which condition it is probable that species do not widely depart, we find that the value 1/3 for the dominance ratio is produced by the asymmetry of the distribution, and in such a manner as to be independent of the activity of the selective agencies, provided that this exceeds a very low level. When differential survival to the extent of only about 1 % in a generation affects the different Mendelian factors, in a population of only a million, and far more for more powerful selection or a larger population, the dominance ratio will be very close to its characteristic value of 1/3*.”

However, this claim is not well-justified theoretically, as shown above. It was criticized by Wright (1931), who pointed out that there was little evidence to support Fisher’s assumption of complete dominance at individual loci affecting quantitative traits. Wright stated that the value 1/3 was based on Fisher’s (1918) use of data on human sibling and parent offspring correlations. He wrote (p. 138): “*It is to be noted, however, that similarity in the environment of brothers as compared with parent and offspring may also contribute to a higher fraternal correlation and that in any case one cannot reason from the dominance ratio deduced from correlations to the distribution of factor frequencies without making some assumption as to the prevalence of dominance*.”

The modern consensus is that quantitative traits such as human body size show a predominance of additive genetic variance, with little evidence for dominance or epistatic contributions (Hill et al. 2008; Visscher and Goddard 2019). However, there is a paradox that has apparently gone unnoticed. The most widely used model for explaining the maintenance of variability in quantitative traits is a balance between new mutations and stabilizing selection, whereby individuals with the extreme values of a trait are selected against (Walsh and Lynch 2018, Chapter 28). Fisher introduced the first population genetic model of this process in Chapter 5 of the *GTNS*, where he assumed that fitness declines as the square of the deviation of the trait value from the optimum (Fisher 1930b).

This model was later analyzed in detail by Wright in 2 influential papers (Wright 1935a, 1935b). Importantly, with additive effects of individual loci on the trait (no dominance), the strength of selection on a given locus is proportional to the product of the measure of the strength of selection on the trait as a whole and the square of the effect of a locus on the trait, *a*_1_^2^ (for a derivation, see Charlesworth and Charlesworth 2010, p. 190). Under the widely accepted “infinitesimal model,” which dates back to Fisher (1918), where most trait variation results from a large number of loci with very small effects, this implies that selection on each locus is very weak, so that variants behave almost neutrally. Wright showed that this means that a large proportion of the genetic variance in fitness (as opposed to the trait itself) is then caused by dominance and epistasis, i.e. it is nonadditive. Most studies have, however, found little evidence for nonadditive variance in traits related to fitness (Charlesworth and Hughes 2000; Charlesworth 2015). It is unclear how this paradox is to be resolved.

## Conclusions

Although Fisher’s belief that the dominance component of the genetic variance of quantitative traits is usually substantial appears to be incorrect, and was due to his neglect of environmental causes of resemblances between relatives, his work introduced most of the basic concepts and methodology used in later research in theoretical population genetics, in an astonishingly profound paper. At the time of writing, he was employed as a statistician at the Rothamsted Experimental Station; he published 7 statistical papers in 1922, including his monumental paper laying the foundations of modern statistical theory (Fisher 1922c), so that his work on population genetics was very much a side show. His analysis of the effects of selection on a single locus anticipated some of the slightly later results of Haldane (Haldane 1924a, 1924b, 1932). Fisher himself seems to have been unaware of the earlier work of the Cambridge mathematician H.T.J. Norton, who provided a table of changes in allele frequencies at loci under selection for Punnett’s book on mimicry (Punnett 1915); this table is reproduced on p. 138 of Provine (1971).

The 1922 paper was the first analysis of stochastic changes in allele frequencies; it led to Fisher’s great 1930 paper, which laid the foundations of the neutral theory of molecular variation, and to Wright’s extensive work on this problem. After 1930, Fisher made only one further original contribution to the theory of random genetic drift: his reexamination of the probability distribution of the frequencies of self-sterility alleles on pp. 104–110 of the second edition of the *GTNS* (Fisher 1958), where he criticized Wright’s pioneering work on this problem (Wright 1939).

Although Fisher consistently discounted the significance of random genetic drift in contributing to evolutionary change, in 1922 he drew the far-sighted conclusion (p. 324) that: “*In all cases it is worth noting that the rate of mutation required* [to maintain a given level of variability] *varies as the variance of this species, but diminishes as the number of individuals is increased. Thus, a numerous species, with the same frequency of mutation, will maintain a higher variability than will a less numerous species: in connection with this fact we cannot fail to remember the dictum of Charles Darwin, that* *‘wide ranging, much diffused and common species vary most’* *(1, chap ii).*”

Fisher strongly emphasized this point at the beginning of Chapter 5 of the *GTNS.*

This view of how the level of variation between individuals within a population is determined is remarkably similar to the modern view of molecular variation in natural populations (Kimura 1983; Walsh and Lynch 2018, Chapter 4), with some important qualifications regarding the effects of selection at linked sites on levels and patterns of variability (Walsh and Lynch 2018, Chapter 8).
